# Peyronie's Disease: Still a Surgical Disease

**DOI:** 10.1155/2012/206284

**Published:** 2012-08-26

**Authors:** Daniel Martinez, Cesar E. Ercole, Tariq S. Hakky, Andrew Kramer, Rafael Carrion

**Affiliations:** 1 Tampa General Circle, Tampa, FL 33606, USA

## Abstract

Peyronie's Disease (PD) remains a challenging and clinically significant morbid condition. Since its first description by François Gigot de la Peyronie, much of the treatment for PD remains nonstandardized. PD is characterized by the formation of fibrous plaques at the level of the tunica albuginea. Clinical manifestations include morphologic changes, such as curvatures and hourglass deformities. Here, we review the common surgical techniques for the management of patients with PD.

## 1. Introduction

Before the times of François Gigot de la Peyronie, men have been plagued with the disfiguring and painful disease eventually known as Peyronie's disease (PD). Curvature develops from the rigid inelastic tunical scar, secondary to macro-/microtrauma in individuals either predisposed genetically or with an underlying disease process of the network of elastic fibers and collagen bundles. This condition causes severe psychological, mental, and physical stress. The pain, erectile dysfunction, and curvature/defect caused by the plaque can prevent proper coitus, potentially resulting in embarrassment and frustration, which may lead to inability to maintain sexual relations. 

Despite the attempts to uncover the pathophysiology behind PD, it still remains an enigma. It has an estimated prevalence of 3–9% although its incidence has increased in recent years [1]. This is partly because men are less embarrassed and more willing to come forth for treatment, rather than silently suffer the pain and difficulties associated with PD.

PD can be characterized by two separate phases. The active (acute) phase is characterized by a painful and evolving plaque, inflammation, and progression of the curvature. This usually lasts 6 to 18 months. Approximately 10% of patients will have improvement in their disease. The majority of patients will experience maintenance or worsening of the defect. Once the disease has been stable for approximately 6 months, this is considered the stable (chronic) phase, at which time surgical treatment is appropriate [2, 3].

In the 18th century, de la Peyronie attempted to treat this ailment by recommending mercurial rubs and bathing in the waters of the River Berges [1, 4]. A multitude of minimally invasive therapies currently exist, including but not limited to, vitamin E (Tocopherol), aminobenzoate potassium (Potaba), colchicine, tamoxifen, intralesional injection therapy with verapamil, interferon, and steroids. Medical treatments have been plagued with flawed results, poorly designed studies, and conflicting data [4]. Surgery remains the mainstay in treatment. However, prior to choosing surgical correction of PD more conservative therapies should have been attempted and failed.

Once the surgeon has determined that the plaque is stable and painless a surgical approach can be taken. Surgical approaches in treating PD have also evolved over time. Table 1 summarizes the current treatments available for PD (Table 1). We review the history and modifications that have been developed, including the classic Nesbit and modified Nesbit operation, penile plication, and incision or excision and grafting. The inflatable penile prosthesis is not reviewed in this paper, but it should be noted that for patients with moderate to severe erectile dysfunction, and complicated plaque defects, the inflatable penile prosthesis either in conjunction with other surgical procedures or as a sole method of therapy is most appropriate.

## 2. Nesbit and Modified Nesbit Operations

In 1965, Nesbit reported his technique for the treatment of congenital chordee [5]. This involved shortening of the longer side of the phallus, thus, the advent of tunical shortening procedures for the treatment of penile curvature was born. Nesbit described making a 5–10 mm transverse elliptical incision of the tunica albuginea on the convex side of the phallus, or approximately 1 mm for each 10° of curvature. This incision was then closed with running nonabsorbable suture.

It was not until 1979 that Pryor and Fitzpatrick applied this technique to the treatment of PD [6]. Since then, this penile shortening technique remains the most popular among urologists, due to its technical simplicity, minimal surgical risk, and quick patient recovery. It has, however, been modified by multiple surgeons. 

Rehman et al. also reported their modification to the classic Nesbit operation [7]. In their approach, they used partial-thickness shaving of the tunica in order to avoid bleeding from the incision site. They would then plicate this area with nonabsorbable suture and buried knots. 

In 1990, Yachia reported his variation to the classic Nesbit operation by incorporating the Heineke-Mikulicz principle [8]. He made a longitudinal incision in the tunica albuginea, followed by a horizontal reapproximation of the edges. 

A number of studies have been performed showing a wide range of patient satisfaction and successful correction of the penile curvature with these tunical shortening surgeries. However, due to the disruption of an intact tunica, and the dissection necessary to expose the area of interest, the Nesbit and Yachia techniques have also been plagued with some degree of erectile dysfunction, increase in patient discomfort, and some reported loss of penile tactile sensation [8]. This led to further modification to the Nesbit procedure and the subsequent development of the Essed-Schroeder technique in 1990. 

Essed and Schroeder introduced the simplest way to surgically treat PD. They described shortening the longer side of the phallus by simple plication with nonabsorbable sutures [9]. Without the need to excise or incise the tunica albuginea or excessive dissection and mobilization of the neurovascular bundle, the hypothetical risks of causing venous leak resulting in erectile dysfunction, or causing loss of penile tactile sensation are decreased. However, this procedure was not without its complications, including tunical tearing by excessive force on the suture, pain from bulky knots, and recurrence of curvature. This led to later modification by Gholami and Lue in 2002 who popularized plication surgery for the treatment of PD, with their 16- or 24-dot minimal tension technique, which is currently the most popular and most performed tunical shortening method for the treatment of PD [10].

## 3. Penile Plication Procedures

With the advent of simple penile plication procedures for the treatment of PD, the armamentarium for treating this condition has grown. The plication technique allows for a rapid and simple surgery, without necessitating dissection of the neurovascular bundle or urethra. It also spares the tunica from being excised or incised, decreasing the morbidity associated with the surgery, and may even be performed under local anesthesia [3].

After the initial introduction of simple penile plication for the treatment of PD pioneered by Essed and Schroeder, there have been a number of modifications to the technique. The initial reports involved shortening the longer side of the tunica albuginea, and applying the necessary amount of stress to the knot required to straighten the phallus, without the need to excise or incise the tunica [9].

In 2002, Gholami and Lue introduced a modification to the original penile plication surgery. Their “16-dot” plication technique allows for distribution of knot tension, making the suture less likely for the suture to tear through the tunica. This also allows less patient discomfort and less episodes of recurrence. They reported that 85% of patients maintained a straight erection over 2.6 years. There was however, some shortening involved, but in only 7% of patients did this cause any functional problems. Twelve percent of patients reported bothersome knots and 11% reported some penile pain with the use of the 2-0 braided, permanent polyester sutures [10].

One of the downsides to the penile plication technique is permanent palpation of the knots, leading to discomfort, focal or erectile pain, and penile induration. This has led to yet another modification, the use of absorbable suture in penile plication surgery. 

In 2001, the concept of using absorbable suture was first introduced. Hsieh et al. reported using 2-0 absorbable polygalactic acid (Vycril) suture for their modified tunical plication technique for the treatment of congenital curvature [11]. In a later article Hsieh reported, 81.5% of patients were either very or moderately satisfied with the surgical outcome, with suture-related complications being very rare. At 6-month follow-up, 86% had straight erections or minimal residual/recurrent curvature, well beyond the 8 weeks that Vycril lasts [12].

**Figure 1:**
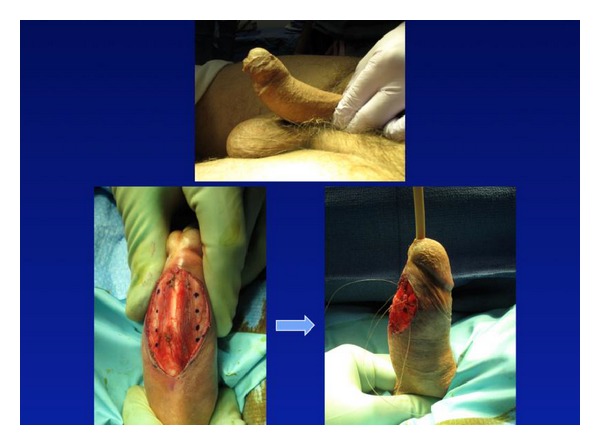


At our institution, we have one of the first series utilizing absorbable suture and longitudinal incisions for the treatment of PD [13] We have incorporated absorbable monofilament 3-0 glycomer (Biosyn) sutures to the 8-dot and 16-dot plication techniques, with resultant correction of the curvature in all patients (Figure 1). In addition to the standard dot plication technique, we favored using longitudinal incisions. In our series of six patients, all report being very satisfied with their surgical results and all have straight erections or minimal recurrent/residual curvatures at 6-month follow-up [13]. A monofilament suture (Biosyn) was used because, as compared with braided suture (Vicryl), the monofilament suture was found to be significantly stronger over 4 weeks of placement, was associated with less local reaction, handled better, and required fewer knots to secure. 

Most critics of absorbable sutures state that they result in a higher rate of curvature recurrence, because of the possibility of plication breakdown after suture material has been reabsorbed. When compared to nonabsorbable suture, absorbable sutures have been shown to result in similar suture failure rates, likely secondary to tissue cut-through of the tunica. Basiri et al. compared plication using absorbable Vicryl suture versus nonabsorbable nylon suture. Both groups had some mild recurrence of curvature and had high-success rates, but patient satisfaction was found to be higher in the Vicryl group because the occurrence of palpable knots was lower in the Vicryl group [14].

Despite multiple advances in penile plication procedures, its applicability to PD is still limited. Those with complex deformities such as hourglass deformities, lateral indentations, or curvatures >60–70° may not be appropriately treated with this technique [2].

Since penile plication is considered a tunical shortening procedure, it is not recommended for patients with shorter phallic lengths. Penile shortening has been reported among 41–90% of patients in the literature, but most patients do not report enough shortening to prevent coitus [3]. In a study conducted by Pryor and Fitzpatrick, they reported the majority of patients having shortening of <1 cm. Only 8.6% reported shortening between 1-2 cm and 4.7% reported >2 cm, but only 1.7% of their patient population reported shortening to the point that precluded sexual intercourse [6]. This possibility leads us to the discussion of the next surgical option for the treatment of PD: the tunical lengthening procedure, and incision or excision of Peyronie's plaque with patch graft.

## 4. Incision or Excision and Patch Graft

Patients with good erectile function with complex curvatures, those with >60° defects, destabilizing hinge defects, and/or shorter phallus, the ideal treatment choice is incision or excision of the plaque and patch grafting. In 1950, Lowsley and Boyce first reported performing plaque excision and grafting with fat for the treatment of PD [15]. Unfortunately, they did not report on follow-up, but this development led the way to the tunical-lengthening procedure for the treatment of PD.

A number of different graft materials have been used over the past decades, and the search for the ideal graft—readily available, pliable, inexpensive, nonthrombogenic, and resistant to infection—has yet to be discovered. Grafts can be divided into three groups: autologous, synthetic, and nonautologous (Table 1). 

Autologous grafts include dermis, vein, tunica vaginalis, temporalis fascia, and buccal mucosa. They have the advantage of causing less inflammatory reaction and lower potential for wound infection as compared with synthetic nonautologous grafts. Unfortunately, autologous grafts are associated with higher surgical morbidity and increased surgical time, because a separate incision has to be made and the graft tissue harvested. This can lead to infection and pain at the graft site.

There have been many studies involving the use of vein grafts for the treatment of PD, especially when patients have more complex anatomical abnormalities. Studies have shown that using a venous graft allows for better elasticity and durability. The vascular endothelium of the graft provides a more physiologically compatible tissue. Usually, the saphenous vein is used because of its ease in harvesting, large surface area providing sufficient length and width, and, when compared to other vein-grafting sites, lower morbidity [16]. Hypothetically, when comparing venous graft (autologous tissues) to synthetic grafts, there are a number of benefits to choosing the former. Synthetic grafts tend to be less elastic, can potentially cause a local inflammatory response, and have a higher potential for wound infection.

Patch grafts for PD using venous tissue have historically had high patient satisfaction and penile straightening rates, upwards into the 90%, especially within the first 12 months. Interestingly, long-term follow-up shows a decrease in satisfaction and straightening. Kadioglu et al., reported their experience with 145 patients, with a mean follow-up of 41.7 months. Only 75.7% reported “completely straightened” penile curvature, while the other 12.8% had less than 20°, and 11.4% reported curvatures >20° residual curvature [17].

Synthetic grafts are no longer recommended because of increased risk of infection, allergic reaction, enhanced inflammation causing fibrosis, and higher rates of contracture [2]. In a study comparing the classic Nesbit, modified Nesbit, and plaque incision and grafting with synthetic grafts, Licht and Lewis reported poor patient satisfaction with synthetic grafts [18].

Nonautologous grafts include pericardium, dermis, fascia lata, dura mater, and porcine dermis. These are divided into two groups: allografts and xenografts. Currently, the two most popular nonautologous xenografts are bovine pericardium and porcine small intestinal submucosa grafts. These two grafts have the advantage of reducing morbidity associated with harvesting of an autologous graft and decreased hypothetical risk of transferring prions and other infectious processes associated with allografts. Serefoglu and Hellstrom compared dermal, pericardial, and small-intestinal submucosal (SIS) grafting, showed similar satisfaction rates and penile curvature correction rates [2]. However, porcine SIS grafts and (human and bovine) pericardium, both, may not be accepted in certain patient populations, for religious reasons.

**Figure 2:**
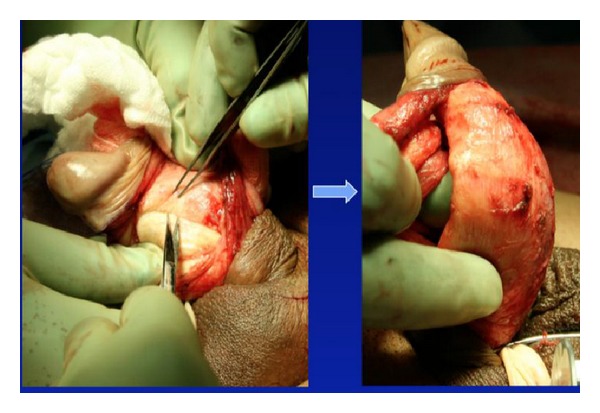


**Figure 3:**
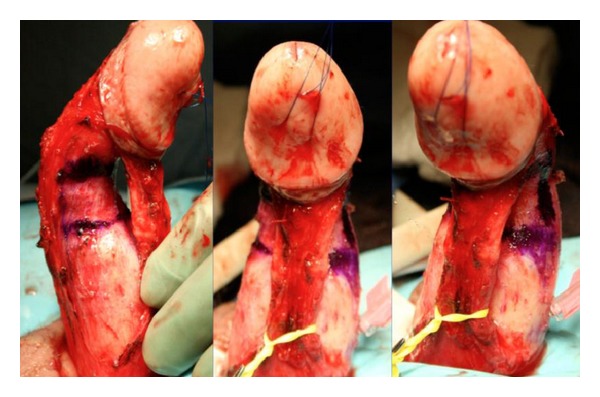


**Figure 4:**
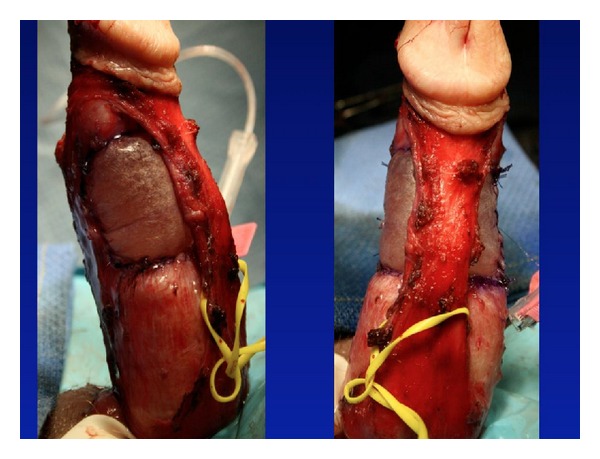


**Figure 5:**
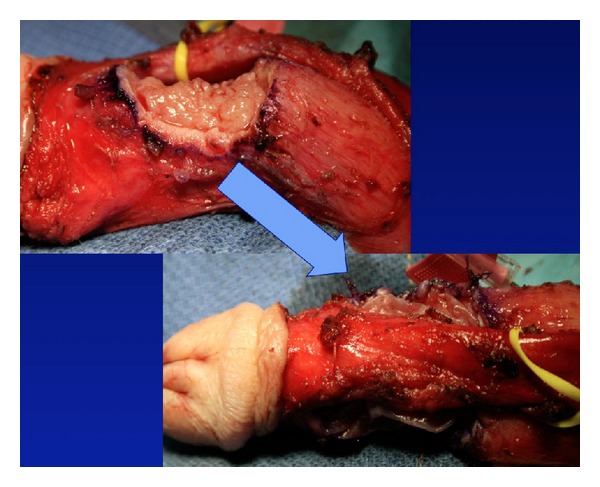


Plaque incision involves initial evaluation in the operating room with an artificial erection, followed typically by a circumcising incision and degloving of the penis. If a ventral plaque is easily accessible via a direct ventral incision, this can be performed longitudinally over the plaque. Next, the neurovascular bundle is dissected off the tunica albuginea within Buck's fascia, with sharp dissection when necessary (Figure 2). Many different incision shapes have been attempted, including but not limited to, an H-shape or “Mercedes-Benz” shape. If larger or calcified plaques are encountered, then an excision of the plaque is performed (Figure 3). The graft is cut 20% larger than the measured defect. The graft is sutured to the tunica albuginea with separate running, locking (or nonlocking) 3-0 or 4-0 polydioxanone suture (Figure 4). An artificial erection is again utilized to assess sufficient correction of the curvature (Figure 5). If necessary, additional plications can be used opposite the graft to improve any residual curvature, or if the curvature is large, a second incision and graft can be utilized. Lastly, Buck's fascia and skin are closed [1].

The “Achilles Heel” of plaque excision or incision and patch grafting has always been worsening erectile dysfunction because of the more extensive dissection and tunical manipulation necessary. For this reason, proper preoperative erectile function evaluation should be undertaken. This includes history and physical, standardized erectile function questionnaires, and possibly intercavernosal injection with Doppler ultrasound examination to assess for arterial insufficiency, venous leak, and evaluation of the plaque. 

Interestingly enough, even with penile lengthening procedures, such as incision or excision and grafting, a proportion of patients still subjectively report a significant decrease in penile length. Some studies report a 35% rate of subjective penile shortening. In 2000, Montorsi et al. objectively reported regarding patient penile length after excision and patch grafting. At 32-month follow-up they noted no change in mean penile length postoperatively, when compared to preoperative length. Regardless, up to 40% of patients still reported subjective shortening [19].

In 1997, Licht and Lewis compared the classic Nesbit, modified Nesbit, and tunical incision and grafting procedures [18]. They showed the greatest amount of satisfaction and lowest erectile dysfunction rate in the group of patients having the modified-Nesbit technique. They also reported the highest rate of phallic shortening in the classic and modified Nesbit groups, but noted that most patients were not bothered by it as long as they were counseled preoperatively [18]. These results are consistent with more contemporary series satisfaction rates that range 67–100% with modified Nesbit procedures [1].

In 2008, Kim et al. reported a study comparing tunical plication versus plaque incision and saphenous vein grafting [20]. At 1-year follow-up there were no statistically significant differences when comparing penile straightening, overall patient satisfaction, erectile pain, and penile shortening in the two groups. The penile plication group did however complain about palpable sutures, but most reported that this was not a significant concern. The incision and graft group reported some loss of sensation. The biggest complaint was loss of erectile rigidity, making intercourse less likely among patients having incision and graft. Operative times also differed greatly, 71 minutes for the plication group versus 234 minutes for the plaque incision and vein-grafting group [20].

## 5. Conclusion

To date, there is no high level of evidence-based data to suggest, which is the best surgical treatment of PD. The perfect treatment choice must be determined by a two-way conversation between the urologist and the patient, keeping in mind the severity of disease, patient preference, and surgeon comfort. There have been studies comparing the different surgical modalities, but the results have not been consistent. A major pitfall includes a lack of standardized training regimens throughout teaching facilities. Moreover, due to the specialized nature of this pathology few providers manage this disease on a high volume basis. All this compounds the outcomes analysis since some reports come from institutions with very little experience managing PD patients. It is our impression that surgical management is currently the only treatment modality to provide acute and satisfactory outcomes to the morphologic deformities associated with PD.

## Figures and Tables

**Table 1:** 

Reconstructive surgery	Prosthesis with or without grafting or molding
Shortening	
Plication, wedge resection	
Lengthening	
Autologus Dermis, tunica vaginalis, buccal mucosa, saphenous vein, temporalis fascia Synthetic Gortex, silastic, Dacron Cadaveric Tutoplast (human pericardium) Surgisis ES (porcine small intestine submucosa) Xenform (acellular bovine dermal matrix)	
